# The Tumor Dynamism Is the Dark Matter of the NGS Galaxy: How to Understand It?

**DOI:** 10.3390/cancers13215476

**Published:** 2021-10-30

**Authors:** Alessandro Ottaiano, Luisa Circelli, Michele Caraglia

**Affiliations:** 1 SSD-Innovative Therapies for Abdominal Metastases Unit, Istituto Nazionale Tumori di Napoli, IRCCS “G. Pascale”, via M. Semmola, 80131 Naples, Italy; 2 AMES, Centro Polidiagnostico Strumentale srl, 80013 Naples, Italy; lulacir@libero.it; 3 Department of Precision Medicine, University of Campania “L. Vanvitelli”, via de Crecchio 7, 80138 Naples, Italy; michele.caraglia@unicampania.it

Since its discovery, there has been a great enthusiasm around NGS (next generation sequencing) technology due to extensive (from restricted gene panels to entire genomes) and rapid (few hours) DNA sequencing [1,2]. This scientific revolution was made possible by integrating biochemistry, molecular technology, and bioinformatics. The potential impact of NGS in oncology research is impressive. In fact, cancer is a complex multi-genic disease, and the identification of genes alterations involved in malignant transformation and progression is pivotal to design innovative and effective therapies. A complete description of the technical and bioinformatics issues (including possible common biases and/or errors, limitations, etc.) to obtain the final genetic variants annotation and prioritization is beyond the scope of this editorial and has been extensively treated elsewhere [3,4,5].

In the large part of experimental settings encountered in the clinical and laboratory practice, researchers have to analyze a cancerous tissue (fresh or paraffin-embedded) selected by the pathologist, in order to be representative of a tumor sample obtained from a surgical resection or a fine-/core-needle biopsy. In fact, the pathologist enriches the analyzed tumor fraction with macro- and micro-dissections by identifying and extracting tumor areas most enriched of cancer cells; the minimum tumor cells content for adequate NGS analysis is typically 20%, particularly when the scope of the analysis is to find specific mutations [6]. When the analysis task is to explore the “genetic landscape” of a tumor either in a single time point or in a comparative perspective (i.e., primary versus metastatic tumor, primary tumors coming from different ethnicities, metastatic lesions coming from different organs, etc.), it is desirable to maintain, as low as possible, the “contamination” of normal cells to avoid interferences or uneven estimations of VAF (variant allele frequency: number of sequence reads of a specific genetic variant/all reads aligned at that locus × 100) of tumor genetic variants. In this context, efforts to obtain >95% of neoplastic cells should be pursued. 

An ideal scenario of 100% neoplastic cells allows us to discuss an actual technical and methodological “dark matter” of all NGS approaches. This dark matter is supported by two strictly related phenomena occurring in cancer: heterogeneity and evolution. In fact, cancer development and progression are prompted and supported by DNA mutations, which are also the fuel of evolution. Humans are, among warm-blooded vertebrates, the most polymorphic species, and their tendency to mutate is both their source of genetic variability and an advantage to evolutionary environment adaptation [7,8]. Unfortunately, cancer arises just as a consequence of these physiologic events (mutations), prompted by an increasingly mutagens pervaded environment. When a tumor establishes, it must adapt to the surrounding environment by genetic evolution. In fact, most malignant cancers progressively acquire and accumulate alterations in genes related to the DNA integrity and stability (i.e., p53, MMR genes, BRCA, BAP1, etc.) [9]. These genetic changes increase cancer mutational plasticity and heterogeneity, making a tumor cell a perfectly dynamic evolutionary machine. Heterogeneity and genetic dynamism (excluding some very rare cases) are constantly present, and crucial for most malignant tumors, and they are responsible for the polyclonal phenotype and for the deadliest phenomena related to cancer: metastases. The environment selects those mutations in a polyclonal tumor population that are more favorable in order to grant survival. Some of these new gained characteristics offer the possibility to escape from immunological control and to migrate from a cancer cell-crowded tissue (poor of nutrient support) to other new unpopulated organs (rich in nutrient support).

A possible scenario of genetic diversity of cancer cells is depicted in Figure 1. In this scenario, a primary tumor is evolving towards a malignant genotype (phenotype), and it acquires a set of metastatic permissive mutations in the red cell. Although fundamental to leave the primary site and spread to distant organs, this neoplastic cell is quantitatively scarcely represented into the primary tumor mass. This accounts for a very low VAF in the final genetic NGS assessment, resulting in a high risk of analytic biases and underestimation. Unfortunately, this is the cell intended for an aggressive neoplastic progeny bearing crucial genetic information. Furthermore, the exclusive analysis of the primary tumor (for healthcare budget limitations and/or unavailability of metastatic tissues) would have completely hidden the genetic alteration underlying the metastasis. In some cases, these genetic variants are found and (wrongly) classified as “metastatic private events”, or with poor significance and penetration. These very crucial early events occurring in primary tumors at unknown time points during the malignant process development are elusive and diluted by a plethora of passenger genetic alterations, as well as polymorphisms.

A single NGS assessment is always a single genetic snapshot. In other words, we take a single photo during a running competition to a single runner with a reduced visual field. It would be better to make a video with a large visual field, to understand the relative position and aspect of each runner, the location, and where they are going to. Liquid biopsy does not lighten into the dark of these elusive crucial events, and it suffers the same quantitative limitations [10,11].

NGS has opened unexpected research scenarios in oncology, allowing large genetic exploration and hypotheses generation. However, cancer does not adapt to technology, but technology must adapt to cancer. An additional improvement of sequencing platforms, technologies (nanopores- or mass spectrometry-based) and bioinformatics is required. In fact, we are assisting the development of new digital high throughput PCR (polymerase chain reaction) platforms, based on the conjugation of PCR techniques with cytofluorometric assays, and of nanotechnology-based biosensors able to detect the presence of mutated circulating tumor DNA directly at the bedside of the patients [12,13,14]. These new technological advancements will allow less invasive, precise (single cell) and dynamic (repeatable) determination of the presence of mutations or other genetic variants in the tumor population from the tissues or blood of the patients (or even from other biological fluids such as saliva and tears). For this reason, the journal *Cancers* has opened a Special Issue to prompt submission of works dealing predominantly with NGS data from colorectal cancer patients. Studies developed through comparative NGS approaches (primary versus metastatic lesions in the same patients from broad or selected clinical settings) or new technological achievements to monitor the dynamic genetic trajectories of cancer and identify the fundamental events of cancer metastatization will be particularly appreciated.

## Figures and Tables

**Figure 1:**
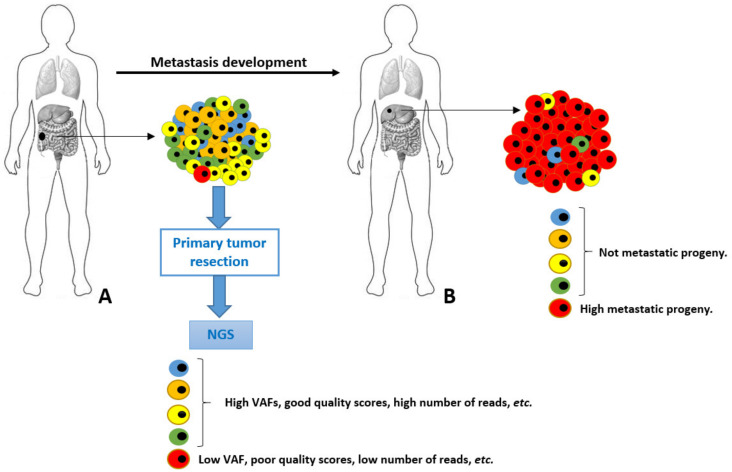
Schematic representation of tumor cells heterogeneity and evolution from primary (**A**) to metastatic lesion (**B**). The highly metastatic red cell in primary tumor is analytically underestimated in NGS performed in **A**.
